# Factors associated with shared decision making among primary care physicians: Findings from a multicentre cross‐sectional study

**DOI:** 10.1111/hex.12603

**Published:** 2017-08-02

**Authors:** Matthew Menear, Mirjam Marjolein Garvelink, Rhéda Adekpedjou, Maria Margarita Becerra Perez, Hubert Robitaille, Stéphane Turcotte, France Légaré

**Affiliations:** ^1^ CHU de Québec Research Centre Quebec QC Canada; ^2^ Department of Family Medicine and Emergency Medicine Laval University Quebec QC Canada; ^3^ Department of Social and Preventive Medicine Laval University Quebec QC Canada

**Keywords:** communication, patient‐centred care, physician‐patient dyads, primary care, shared decision making

## Abstract

**Background:**

Despite growing recognition that shared decision making (SDM) is central for patient‐centred primary care, adoption by physicians remains limited in routine practice.

**Objective:**

To examine the characteristics of physicians, patients and consultations associated with primary care physicians’ SDM behaviours during routine care.

**Methods:**

A multicentre cross‐sectional survey study was conducted with 114 unique patient‐physician dyads recruited from 17 primary care clinics in Quebec and Ontario, Canada. Physicians’ SDM behaviours were assessed with the 12‐item OPTION scale scored by third observers using audio‐recordings of consultations. Independent variables included 21 physician, patient and consultation characteristics. We assessed factors associated with OPTION scores using multivariate linear regression models.

**Results:**

On the OPTION scale, where higher scores indicated greater SDM behaviours, physicians earned an overall mean score of 25.7±9.8 of 100. In the final adjusted regression model, higher OPTION scores were associated with physicians’ social participation (involvement in one committee β=5.75, *P*=.04; involvement in two or more committees β=7.74, *P*=.01), patients’ status as employed (β=6.48, *P*=.02), clinically significant decisional conflict in patients (β=7.15, *P*=.002) and a longer duration of consultations (β=0.23, *P*=.002).

**Conclusion:**

Physicians’ social participation, patients’ employment status and decisional conflict and the duration of consultations were associated with primary care physicians’ SDM behaviours in routine care. These factors should be considered when designing strategies to implement SDM and promote more patient‐centred care in primary care.

### 1 | INTRODUCTION

The idea of patient-centred care has become a central tenet of health-care systems worldwide, and attention is now turning to how make the delivery of such care a practical reality.[1] Considered to be the crux of patient-centred care,[2,3] the promotion of shared decision making (SDM) appears to be a key strategy in this endeavour. SDM is a process by which health decisions are deliberated upon and made jointly by the patient and one or more health professionals, taking into consideration the best available evidence and the patients' values and preferences.[4-6] Systematic reviews suggest that SDM and SDM tools (e.g, decision aids) support patient-centred care by improving patient-professional communication and patients' knowledge of their options, their levels of trust and satisfaction with providers and their sense of control over medical decisions and problems.[7,8] Given the increasingly strong evidence in favour of SDM, a growing number of countries are incorporating SDM within their health-care policies and reform initiatives.[9-11]

While some of its roots can be traced to specialty care, much of the pioneering research and evidence on SDM has been produced in primary care contexts.[12-17] Indeed, primary care offers fertile ground for innovation in decision making given the sheer volume of service use in these settings, the spectrum of health conditions encountered and the great diversity of medical decisions made daily.[18-20] In addition, relationships between primary care physicians and their patients can span years and even decades, enabling a foundation of understanding, trust and respect that can greatly facilitate SDM.[20,21] Yet, despite the accumulating evidence for the benefits of SDM and increased contextual pressures for its implementation in primary care,[11] many physicians have still not adopted SDM as part of their routine primary care practice.[22,23]

It is currently unclear what factors contribute to greater or lesser SDM behaviours by primary care physicians. A systematic review of health professionals' perceived barriers and facilitators to SDM implementation identified time pressures, characteristics of patients and clinical situations, professionals' familiarity with patients, and patients' preferences and preferred role in decision making as among the most frequently cited factors influencing patient involvement in SDM.[24] However, these results stem mostly from qualitative studies conducted in a variety of clinical settings. To date, studies specifically investigating the SDM behaviours of primary care physicians have been largely descriptive in nature[23] and only a few have sought to identify factors influencing physicians' SDM levels. Furthermore, the findings of these latter studies are inconsistent and largely limited by small sample sizes,[25,26] the inclusion of a small number of predictor variables,[27-29] unadjusted analyses[25,26,28-30] or a focus only on physicians in training.[31]

A better understanding of the factors associated with SDM among primary care physicians could support more targeted efforts to implement SDM and promote more patient-centred practices in primary care. This study thus aimed to examine the characteristics of physicians, patients and consultations associated with primary care physicians' SDM behaviours during routine care.

### 2 | METHODS

#### 2.1 | Study design and participants

Data were obtained from the EXACKTE[2] study, a multicentre cross-sectional survey study conducted in two Canadian provinces.[18,32] The EXACKTE[2] study is distinct in its enrolment of a large number of unique patient-physician dyads.[32,33] Briefly, licensed and active primary care physicians were recruited from six family medicine clinics in Quebec City, Quebec, and 11 primary care clinics in London, Ontario. After consenting to participate, physicians granted us permission to recruit their patients in the waiting room at random moments during physicians' visiting hours. One patient was recruited per physician, thus forming the unique dyad. Patients were eligible if they were ≥18 years old, able to read English or French, able to provide informed consent, not suffering from an acute condition requiring urgent medical attention (i.e, transfer to emergency department) and willing to report on a health decision they made with their physician.

#### 2.2 | Data collection

Prior to patient recruitment, physicians completed a consent form and a questionnaire assessing their general attitudes towards uncertainty and their professional and socio-demographic characteristics. Following patient recruitment, we audiotaped their encounters with physicians to evaluate the extent of SDM that occurred. After the consultation, physicians completed a questionnaire assessing the decision that was made and their decisional conflict during the consultation. Patients were also surveyed after the consultation to collect data on the decision that was made, their general preferred role in decision making, the conflict they had about the decision and their socio-demographic characteristics. Information on the specific decision made was confirmed using the tape recordings of consultations.

#### 2.3 | Dependent variable—Shared decision making behaviours

Physicians' SDM behaviours were assessed using the third-observer OPTION ("observing patient involvement in decision making") scale.[34] The OPTION scale has been the most prominent measure of SDM used in health-care research,[35] and its scores are obtained from the perspective of external observers based on routine consultations. The scale consists of 12 items, which were rated by four trained coders (at least two per consultation) using the verbatim transcripts of clinical consultations. Coders rated each item on a 5-point scale from 0 to 4, with a score of 0 indicating that the specific SDM-related behaviour was not observed, a score of 1 indicating that a minimal attempt was made to exhibit the behaviour and a score of 4 indicating that the behaviour was observed and executed to a high standard. The intraclass correlations for the scores of the four raters varied between 0.64 (95% CI=0.44-0.82) and 0.88 (95% CI=0.76-0.95), suggesting adequate inter-rater agreement. We then calculated an overall score ranging from 0 (no SDM behaviours) to 100 (optimal SDM behaviours)

by summing the item scores, dividing by 48 (the maximum total raw score), and multiplying by 100. An overall score of 25 (or 1 on each item) suggests at least minimal competence in involving patients in SDM.[34]

### 2.4 | Independent variables

We investigated associations between physicians' SDM behaviours and three types of factors: physician characteristics, patient characteristics and characteristics of consultations. *Physician characteristics* included socio-demographics such as age and sex and professional characteristics such as years of clinical experience. Selection of additional characteristics was informed by Rogers' Diffusion of innovations theory.[36] According to this theory, early adopters of innovations have more formal education, more social participation and cope better uncertainty than later adopters. Differences in years of formal education were thus captured by asking physicians if they had completed an academic degree prior to their medical training. Social participation relates to individuals' opportunities to communicate face-to-face with others, discuss new ideas, be exposed to influential opinion leaders and gain access to social networks. We assessed physicians' social participation through a question about the number of committees (e.g, institutional, academic, regional and professional) they were involved in over the past year. We collected data related to physicians' ability to cope with uncertainty using the Physicians' Reactions to Uncertainty scales developed by Gerrity, which examine general anxiety due to uncertainty and reluctance to disclose uncertainty to patients.[37] Given our study's objectives, we also assessed physicians' decisional conflict using the 16-item clinician version of the Decisional Conflict Scale (DCS).[38]

*Patient characteristics* included their age, sex, marital status, education, employment status, income, preferred role in decision making and decisional conflict. Patients' generally preferred role in decision making was assessed by a single question with five response options: (i) clinician makes the decision; (ii) clinician makes the decision, but strongly considers patient opinion; (iii) clinician and patient make the decision together, on an equal basis; (iv) patient makes the decision, but strongly considers clinician opinion; and (v) patient makes the decision.[12] Conflict about the decision they had to make during the consultation was assessed using the 16-item patient version of the DCS.[39]

*Characteristics of consultations* included the patient's familiarity with the doctor (self-reported, first-time visit or not), sex concordance between patients and physicians (i.e, same sex, different sex), type of decision made, length of the consultation (in minutes) and province in which care is delivered. The specific health decision made was allowed to vary broadly and was self-reported by patients and confirmed by physicians. These decisions were categorized thematically into types based on previous work by Légaré and colleagues.[40]

### 2.5 | Data analysis

We performed descriptive analyses on physician, patient and consultation characteristics and OPTION scores using means, medians, standard deviations, minimums/maximums and interquartile ranges for continuous variables and percentages for categorical variables. We reported scores for each OPTION item and calculated a global OPTION score of 100. The normality of the distribution of scores on all continuous variables was examined using Shapiro-Wilk tests and visual inspection (e.g, histograms, normal-quantile plot, qq plot). Normality was confirmed for all variables except for decisional conflict scores, leading us to dichotomize decisional conflict scores using a cut-off point recognized in the literature to distinguish clinically significant decisional conflict from less significant conflict (scores ≥25 of 100).[41] Next, we calculated an intraclass correlation to verify the province-level effects on OPTION score variability, which were negligible (ICC=0.03, $P$=.33). We then performed bivariate analyses to examine relationships between independent variables and global OPTION scores. We made only one a priori hypothesis based on the literature[23] that SDM behaviours would be positively associated with the length of consultations. Following bivariate analyses, due to appropriate assumptions, we performed multivariate linear regression analyses using both bidirectional and backward elimination. Multicollinearity between variables was verified, leading us to drop the variable related to physician experience from analyses due to its correlation with physician age. We also dropped variables related to physicians' and patients' sex, choosing to keep instead the variable on physician-patient sex concordance given a previous study that showed a significant association between gender dyads and OPTION scores.[26] A $P$-value of <.10 was used as the threshold for statistical significance in the final model due to the exploratory nature of the model. However, we also considered adjusted-$R^2$ when making choices about independent variables to include in our models. All statistical analyses were performed with SAS version 9.4.

The EXACKTE[2] study was funded by the Canadian Institutes of Health Research (CIHR grant #185649 KTE). The CIHR had no involvement in this study's design, data collection or analysis, or interpretation and reporting of results.

## 3 | RESULTS

### 3.1 | Characteristics of participants and consultations

A total of 170 eligible licensed primary care physicians were approached to participate in the EXACKTE[2] study. Of these, 117 (69%) agreed to participate and completed our questionnaires. An equal number of patients were recruited, with 114 unique patient-physician dyads providing complete data for the study (Figure 1). The majority of dyads were recruited in Quebec and represented patients consulting physicians they had met with before. The full list of characteristics of participants and consultations are provided in Table 1.

### 3.2 | Shared decision making behaviours

With respect to physicians' SDM behaviours, physicians earned an overall mean score of 25.7±9.8 of 100 (median 25.0, range 6.25

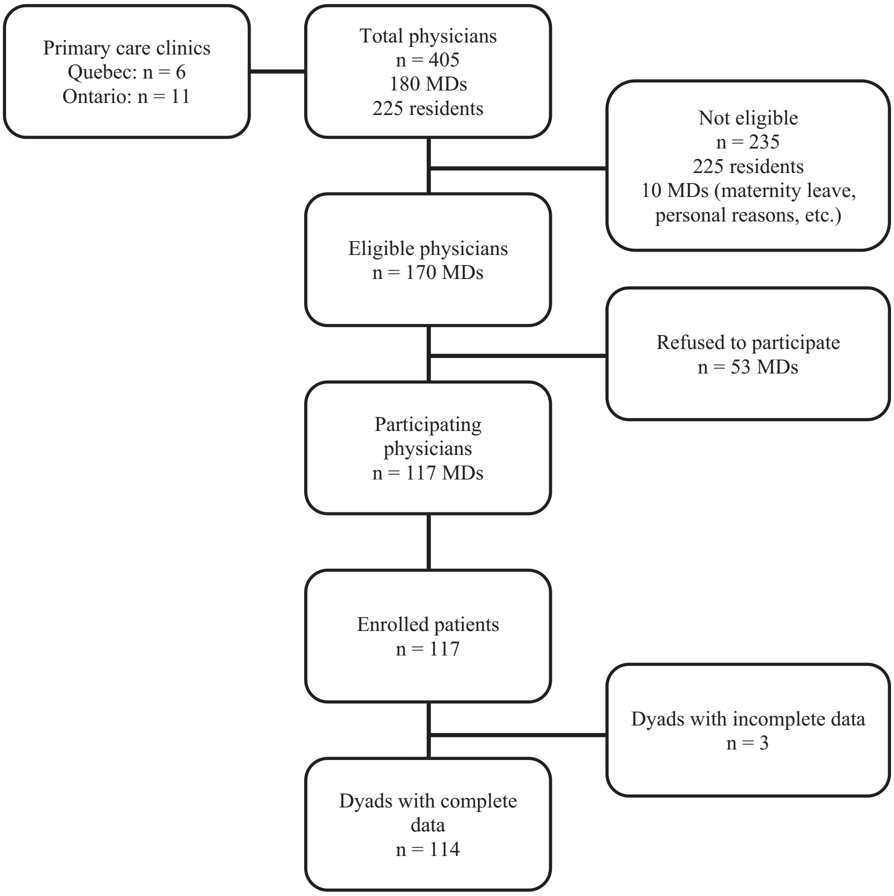

**FIGURE 1** Flow diagram of physician-patient dyad recruitment

- 52.08) on the OPTION scale. Mean scores by item ranged between 0.15/4 and 1.57/4 (Table 2). The two highest scoring items were "The clinician lists options, which can include the choice of no action" (1.57/4) and "The clinician draws attention to an identified problem as one that requires a decision making process" (1.50/4). Four items had mean scores below 1 (items 2, 3, 10 and 11; Table 2).

#### 3.3 | Factors associated with shared decision making

In bivariate analyses (Table 3), physicians' decisional conflict, patients' employment status and decisional conflict, patients' familiarity with the physician, the type of decision and the duration of consultations were associated with physicians' OPTION scores. In multivariate analyses (Table 3), the characteristics associated with higher OPTION scores among primary care physicians were physicians' greater social participation (involvement in one committee $\beta$=5.75, *P*=.04; involvement in two or more committees $\beta$=7.74, *P*=.01), clinically significant decisional conflict in patients ($\beta$=7.15, *P*=.002) and a longer duration of consultations ($\beta$=0.23, *P*=.002) (Table 3). Thus, physicians' SDM behaviours were significantly greater when they were involved in some committee work (with scores higher when involved in two or more committees), when patients reported significant decisional conflict (scores of 25 or more on the DCS) and when consultations with patients were longer. There was also a negative association between physicians' OPTION scores and patients' employment status, such that scores were significantly lower when patients were unemployed relative to being employed ($\beta$=- 6.48, *P*=.02). Finally, there was a trend for increased OPTION scores as the age of physicians decreased. The variance explained by the final model was 23%.

### 4 | DISCUSSION

We examined SDM behaviours among primary care physicians and aimed to identify the physician, patient and consultation characteristics associated with these SDM behaviours during routine care. We found that physicians performed only minimal behaviours to involve patients in decisions about their care. Our novel finding was that four variables covering all three types of characteristics were found to be associated with physicians' SDM behaviours, namely physicians' social participation, patients' employment status and decisional conflict and the duration of consultations. Such associations remained significant after controlling for other physician-, patient- and consultation-level factors.

Our findings reinforce the idea that concerted efforts are needed to improve the implementation of SDM in primary care. The relatively low OPTION scores we observed among physicians were highly consistent with a large number of descriptive studies on SDM in primary care. Much of this evidence was recently synthesized in a recent systematic review of studies on SDM behaviours in health care.[23] The

**TABLE 1** Participant and consultation characteristics (n=114 dyads)

| Characteristics | N (%) or mean±SD |
|---|---|
| Physicians | |
| Age (years) | 45.3±9.5 |
| Experience (years) | 17.8±10.8 |
| Sex | |
| Female | 64 (56.1) |
| Male | 50 (43.9) |
| Completed an academic degree prior to medical training | |
| No | 43 (37.7) |
| Yes | 71 (62.3) |
| Committee involvement in past year | |
| None | 16 (14.0) |
| One committee | 39 (34.2) |
| Two or more committees | 59 (51.8) |
| Decisional conflict score (DCS) | |
| DCS=0-<25 | 57 (50.0) |
| DCS=≥25-100 | 56 (49.1) |
| Missing | 1 (0.9) |
| Physician anxiety towards uncertainty (range 5-30)[a] | 14.8±4.7 |
| Physician reluctance to disclose uncertainty (range 5-24)[a] | 11.6±5.1 |
| Patients | |
| Age (years)[a] | 53.5±17.3 |
| Sex | |
| Female | 91 (79.8) |
| Male | 23 (20.2) |
| Marital status | |
| Married | 83 (72.8) |
| Not married (i.e, single, divorced, widowed) | 31 (27.2) |
| Education | |
| High school or less | 35 (30.7) |
| College/Professional | 35 (30.7) |
| University | 42 (36.8) |
| Missing | 2 (1.8) |
| Employment status | |
| Employed (full time or part-time) | 59 (51.8) |
| Unemployed or other (e.g, at home, on disability) | 12 (10.5) |
| Retired | 43 (37.7) |
| Income[b] | |
| 0-49 K | 37 (32.5) |
| 50-59 K | 13 (11.4) |
| 60-79 K | 21 (18.4) |
| 80-100 K | 18 (15.8) |
| >100 K | 17 (14.9) |
| Missing | 8 (7.0) |

(Continues)

**TABLE 1** (Continued)

| Characteristics | N (%) or mean±SD |
|---|---|
| Preferred role in decision making | |
| Clinician makes the decision, with or without considering patient's opinion | 38 (33.3) |
| Clinician and patient make the decision together, on an equal basis | 54 (47.4) |
| Patient makes the decision, with or without considering clinician's opinion | 20 (17.5) |
| Missing | 2 (1.8) |
| Decisional conflict score (DCS) | |
| DCS=0-<25 | 89 (78.1) |
| DCS=≥25-100 | 20 (17.5) |
| Missing | 5 (4.4) |
| Consultations | |
| Province | |
| Quebec | 74 (64.9) |
| Ontario | 40 (35.1) |
| Familiarity with physician | |
| First time consulting the physician | 8 (7.0) |
| Has consulted the physician before | 106 (93.0) |
| Physician-patient sex concordance | |
| Same sex | 65 (57.0) |
| Different sex | 49 (43.0) |
| Decision type | |
| Monitoring, follow-up and referrals | 24 (21.1) |
| Lifestyle | 7 (6.1) |
| Diagnostic and screening tests | 25 (21.9) |
| Treatment related | 51 (44.7) |
| Other (e.g, birth control, vaccination, return to work) | 7 (6.1) |
| Length of consultation (minutes) | 27.6±12.4 |

[a]Missing values: physician anxiety towards uncertainty (2), physician reluctance to disclose uncertainty (2), patient age (1).
[b]K=1000$ (Canadian dollars).

authors found 33 studies using the OPTION scale to assess SDM, including 16 studies with primary care physicians. Among these 16 studies, only five reported mean OPTION scores greater than 25 out of 100 and in only one study was the mean score above 35, lower scores than were observed in studies with medical specialists and non-physicians. Such results beg the question: What factors may be positively or negatively influencing primary care physicians' SDM behaviours?

While some studies have reported associations between primary care physicians' age or gender and their SDM behaviours,[25,26] the only physician characteristic associated with OPTION scores in the current study was physicians' involvement in committee work. Based on Rogers' theory on innovation,[36] we treated committee work as a proxy for active physician participation in their social system, which is thought to lead to greater interconnectedness with others, exposure to new ideas and likelihood of adopting innovations. That associations

**TABLE 2** Physicians' scores on OPTION scale, by item and total score (n=114)

| | Mean score | SD | Minimum score | Maximum score |
|---|---|---|---|---|
| 1. The clinician draws attention to an identified problem as one that requires a decision-making process | 1.50 | 0.65 | 0 | 4 |
| 2. The clinician states that there is more than one way to deal with the identified problem (equipoise) | 0.43 | 0.58 | 0 | 3 |
| 3. The clinician assesses the patient's preferred approach to receiving information to assist decision making (e.g, discussion, reading printed material, assessing graphical data, using videotape or other media) | 0.15 | 0.40 | 0 | 2 |
| 4. The clinician lists options, which can include the choice of "no action" | 1.57 | 0.81 | 0 | 3 |
| 5. The clinician explains the pros and cons of options to the patient (taking "no action" is an option) | 1.40 | 0.71 | 0 | 3 |
| 6. The clinician explores the patient's expectations (or ideas) about how the problem(s) is to be managed | 1.19 | 0.75 | 0 | 3 |
| 7. The clinician explores the patient's concerns (fears) about how the problem(s) is to be managed | 1.11 | 0.80 | 0 | 3 |
| 8. The clinician checks that the patient has understood the information | 1.06 | 0.65 | 0 | 3 |
| 9. The clinician offers the patient explicit opportunities to ask questions during the decision-making process | 1.27 | 0.63 | 0 | 3 |
| 10. The clinician elicits the patient's preferred level of involvement in decision making | 0.79 | 0.80 | 0 | 4 |
| 11. The clinician indicates the need for a decision-making (or deferring) stage | 0.83 | 0.81 | 0 | 3 |
| 12. The clinician indicates the need to review the decision (or deferment) | 1.05 | 0.69 | 0 | 2 |
| Total OPTION score (0-48) | 12.34 | 4.70 | 3 | 25 |

were strongest when physicians were involved in multiple committees could also be considered consistent with Rogers' views tying innovation to more cosmopolitan perspectives, that is, looking beyond their local situation for guidance and work satisfaction.[36,42] While encouraging physicians to take on greater committee work may not be a realistic strategy in many clinical settings, clinical leaders could explore other options (e.g, regional conferences, journal clubs, clinical communities of practice) for enhancing physicians' social participation and connectedness with others as a way to facilitate local adoption of innovations such as SDM.

Two patient characteristics were related to primary care physicians' SDM behaviours. To our knowledge, we are the first study to report an association between SDM and primary care patients' employment status. Specifically, we found that physicians' OPTION scores were lower in consultations with unemployed patients relative to patients that were employed. Surprisingly, no significant associations between SDM behaviours and patients' income or education levels were observed. Previous work has shown that primary care patients from lower socio-economic backgrounds adopt more passive roles in decision making during their consultations with physicians,[43] and this may have been the case with the unemployed patients in our study. Another possibility is that physicians made assumptions about which patients may prefer or benefit from SDM, ultimately deciding that SDM was less applicable when patients were unemployed with a potentially lower socio-economic status.[24] Such misconceptions are problematic as vulnerable patient groups may actually be the ones that stand to benefit the most from SDM and the reluctance that these patients sometimes show in engaging in decision making reflects not a lack of interest but rather low self-efficacy for greater engagement.[44] Thus, while physicians have little control over the employment status of their patients, actions to promote SDM can target physicians' attitudes and the better engagement of socially vulnerable populations in care decisions.

Similar to patients' employment status, we are the first study to find a positive association between clinically significant decisional conflict in patients and primary care physicians' OPTION scores. Decisional conflict is common in primary care[41] and occurs when people are uncertain about a decision, especially when that decision involves potentially significant risks, losses or regrets.[45] Higher patients' decisional conflict has been shown to manifest itself as increased worrying and nervousness about decisions[46] and may lead patients to verbalize their distress and uncertainty about options to physicians,[47] thus prompting greater efforts on their part to engage the patient in SDM. Unfortunately, the OPTION scale has a physician-centric scoring approach and it is unclear how patients' actions may have influenced physicians' SDM behaviours in our study. While decisional conflict is sometimes portrayed as having negative effects on patient outcomes, our study indicates that it might also be a facilitator or by-product of more in-depth physician-patient conversations on options and potential benefits and harms of these options.[48]

Consistent with our only a priori hypothesis, we found that longer consultations were strongly associated with physicians' SDM behaviours. This relationship has been observed in previous studies with primary care physicians[28,29,31] as well as health-care providers generally.[23] As argued by several authors,[30,49] SDM is a process and achieving full participation of patients in health decisions takes

**TABLE 3** Factors associated with primary care physicians' OPTION scores

| Characteristics | Bivariate analyses β | SE | P-value | Final model[a] β | SE | P-value |
|---|---|---|---|---|---|---|
| Physician characteristics | | | | | | |
| Age (years) | -0.14 | 0.10 | .14 | -0.18 | 0.10 | .07 |
| Degree prior to medical training | | | | | | |
| Yes (vs No) | 1.24 | 1.90 | .51 | | | |
| Committee involvement | | | | | | |
| One (vs None) | 3.45 | 2.90 | .24 | 5.75 | 2.82 | .04 |
| Two or more (vs None) | 4.48 | 2.75 | .11 | 7.74 | 2.75 | .01 |
| Decisional conflict[b] | | | | | | |
| DCS=≥25-100 (vs DCS=0-<25) | 3.27 | 1.83 | .08 | | | |
| Anxiety towards uncertainty | 0.07 | 0.20 | .72 | | | |
| Reluctance to disclose uncertainty | -0.12 | 0.18 | .50 | | | |
| Patient characteristics | | | | | | |
| Age (years) | 0.0004 | 0.05 | .99 | | | |
| Marital status | | | | | | |
| Not married (vs Married) | 2.18 | 2.06 | .29 | | | |
| Education | | | | | | |
| College/Professional degree (vs Secondary or none) | -2.11 | 2.32 | .37 | -1.79 | 2.17 | .41 |
| University degree (vs Secondary or none) | 2.14 | 2.22 | .34 | 3.05 | 2.13 | .15 |
| Employment status | | | | | | |
| Unemployed or other (vs Employed) | -6.37 | 3.06 | .04 | -6.48 | 2.83 | .02 |
| Retired (vs Employed) | -2.22 | 1.94 | .26 | -1.15 | 1.88 | .54 |
| Income[b] | | | | | | |
| 50-59 K (vs 0-49 K) | 3.25 | 3.17 | .31 | | | |
| 60-79 K (vs 0-49 K) | 1.59 | 2.69 | .56 | | | |
| 80-100 K (vs 0-49 K) | -0.22 | 2.83 | .94 | | | |
| >100 K (vs 0-49 K) | 0.62 | 2.88 | .83 | | | |
| Preferred role in decision making | | | | | | |
| Clinician makes the decision (vs Shared decision) | -2.16 | 2.09 | .30 | | | |
| Patient makes the decision (vs Shared decision) | 0.61 | 2.59 | .81 | | | |
| Decisional conflict[b] | | | | | | |
| DCS=≥25-100 (vs DCS=0-<25) | 5.17 | 2.42 | .03 | 7.15 | 2.28 | .002 |
| Characteristics of consultations | | | | | | |
| Familiarity with physician | | | | | | |
| First-time consulting (vs Has consulted before) | 6.81 | 3.55 | .06 | | | |
| Physician-patient sex concordance | | | | | | |
| Different sex (vs Same sex) | 1.55 | 1.85 | .40 | | | |
| Type of decision[c] | | | | | | |
| Lifestyle (vs Monitoring, follow-up, referrals) | 3.92 | 4.21 | .35 | | | |
| Diagnostic and screening tests (vs Monitoring, follow-up, referrals) | 1.93 | 2.80 | .49 | | | |
| Treatment related (vs Monitoring, follow-up, referrals) | 5.13 | 2.43 | .04 | | | |
| Other (vs Monitoring, follow-up, referrals) | 5.69 | 5.30 | .29 | | | |

(Continues)

**TABLE 3** (Continued)

| Characteristics | Bivariate analyses β | SE | *P*-value | Final model[a] β | SE | *P*-value |
|---|---|---|---|---|---|---|
| Length of consultations (minutes) | 0.24 | 0.07 | .001 | 0.23 | 0.07 | .002 |
| Explained Variance, Adjusted $R^2$ | | - | | | 0.22 | |

[a]Model with selection of variables according to backward procedure ($\alpha_{OUT}$=0.10); the variables related to physicians' age and committee involvement and patients' education were not significant at alpha <0.10; however, these variables were kept in the model because they explained between 4% and 6% of the variance. β=Beta coefficient, SE=Standard error.
[b]DCS=Decisional Conflict Scale, K=1000$ (Canadian dollars).
[c]Other=Decisions related to vaccinations, birth control, return to work.

time. From both clinical and policy perspectives, the question then becomes, how much time is needed to enable SDM in routine primary care? Based on her work in varied clinical settings, Kaplan hypothesized that visits of at least 20 minutes may be needed.[49] Visits of this duration may be particularly appropriate when it is clear that preference-sensitive decisions need to be made in which the best option for patients is not clear.[50] In such circumstances, sufficient time is needed to clarify the decision, discuss the available options and their pros and cons, explore patients' preferences and concerns, and arrive at a decision together. In many primary care practices where visits frequently last less than 10 minutes,[51] physicians' ability to deliberate with patients in this way is limited, leading some to feel that practicing SDM is inconsistent with the time pressures of their clinical reality.[24] However, it should be recognized that other professionals can be involved in supporting decision-making processes, thus resulting in an interprofessional approach to SDM.[5] Furthermore, the steps in SDM may be spread out among multiple consultations, allowing more time for patients to reflect on options and consider their preferences and values. Interestingly, the mean consultation length in the present study was nearly 28 minutes and yet physicians' overall SDM competence remained low. Thus, while professionals often perceive time constraints as the main barrier to SDM adoption,[24] clearly, other factors contributed to the minimal SDM behaviours of physicians in our study.

Several limitations of our study should be noted. First, SDM is a complex process that likely requires a longitudinal approach to better understand how decision making unfolds over time. Given our assessment of single consultations, it is possible that we have not fully captured the SDM processes that occurred between patients and their physicians. For some patients, it is possible that more important or more preference-sensitive decisions were made during visits other than the one being examined as part of this study and that more SDM behaviours occurred during these other consultations. Second, the 12-item OPTION scale has also been critiqued for including some items related to idealized behaviours that rarely occur in practice (e.g, item 3), contributing to floor effects.[52] In our study, however, physicians still scored poorly on core SDM behaviours, including listing options, explaining pros and cons and offering patients opportunities to ask questions and provide input during the decision-making process. High scores on the OPTION scale are achievable, as demonstrated by a study on primary care physicians in the United Kingdom that were already experienced in SDM.[53] Third, all of the family medicine clinics involved in this study were academic sites involved in teaching and research. The characteristics of these sites can differ from other private primary care clinics, which could limit the generalizability of our findings. Finally, this study was exploratory in nature and examined relationships between a range of physician, patient and consultation characteristics and physicians' SDM behaviours. However, the variance explained by our final model was low (23%), suggesting that there are likely other factors out there that influenced physicians' SDM behaviours but that remained unidentified. Factors that might be explored in future studies include physicians' preferences and training for SDM, physicians' communication skills, patients' communication skills and self-efficacy for involvement in decision making, the nature and number of patients' health problems, the urgency and sensitivity of the decision, environmental conditions for decision making (e.g, noise, privacy), trust between patients and physicians, and whether patients were prepared for the encounter and for SDM.[36,54,55]

### 5 | CONCLUSION

This study found that primary care physicians' SDM behaviours were associated with the characteristics of the participants in SDM (physicians' social participation, patients' employment status and decisional conflict), as well as a consultation characteristic tied to the interaction between SDM participants (consultation length). These factors should be considered when designing strategies to implement SDM in primary care, and several practical ways of doing so are mentioned in the discussion. At the same time, our results also highlight that there remains much to learn about the forces that facilitate and impede physicians' SDM behaviours. There is a clear need to identify novel factors related to physicians, patients and practice contexts that help explain why SDM does or does not occur in routine care. This is an important knowledge gap that warrants attention to further advance patient-centred care practices in primary care.

#### ACKNOWLEDGEMENTS

The authors would like to thank all the physicians and patients who participated in the study, the recruitment teams in London and

Quebec City and the coders who rated the consultations with the OPTION scale (Véronique Couture, Sébastien Courchesne-O'Neill, Hubert Robitaille and Marc-André Pellerin).

### CONFLICT OF INTERESTS

The authors declare no conflict of interests.
